# Sun Exposure and Vitamin D Supplementation in Relation to Vitamin D Status of Breastfeeding Mothers and Infants in the Global Exploration of Human Milk Study

**DOI:** 10.3390/nu7021081

**Published:** 2015-02-05

**Authors:** Adekunle Dawodu, Barbara Davidson, Jessica G. Woo, Yong-Mei Peng, Guillermo M. Ruiz-Palacios, Maria de Lourdes Guerrero, Ardythe L. Morrow

**Affiliations:** 1 Global Health Center, Perinatal Institute and Division of Biostatistics and Epidemiology, Cincinnati Children’s Hospital Medical Center, 3333 Burnet Avenue, Cincinnati, OH 45229, USA; E-Mails: barbara.davidson@cchmc.org (B.D.); jessica.woo@cchmc.org (J.G.W.); ardythe.morrow@cchmc.org (A.L.M.); 2 Children’s Hospital of Fudan University, 399 Wanyuan Road, Shanghai 20102, China; E-Mail: ympeng@fudan.edu.cn; 3 National Institute of Medical Sciences and Nutrition, Vasco de Quiroga No. 15, Tlalpan, Mexico City 14000, Mexico; E-Mails: gmrps@ unam.mx (G.M.R.P.); mlga08@gmail.com (M.L.G.)

**Keywords:** vitamin D, sun exposure, breastfeeding, supplements, mothers, infants

## Abstract

Although vitamin D (vD) deficiency is common in breastfed infants and their mothers during pregnancy and lactation, a standardized global comparison is lacking. We studied the prevalence and risk factors for vD deficiency using a standardized protocol in a cohort of breastfeeding mother-infant pairs, enrolled in the Global Exploration of Human Milk Study, designed to examine longitudinally the effect of environment, diet and culture. Mothers planned to provide breast milk for at least three months post-partum and were enrolled at four weeks postpartum in Shanghai, China (*n* = 112), Cincinnati, Ohio (*n* = 119), and Mexico City, Mexico (*n* = 113). Maternal serum 25(OH)D was measured by radioimmunoassay (<50 nmol/L was categorized as deficient). Serum 25(OH)D was measured in a subset of infants (35 Shanghai, 47 Cincinnati and 45 Mexico City) seen at 26 weeks of age during fall and winter seasons. Data collected prospectively included vD supplementation, season and sun index (sun exposure × body surface area exposed while outdoors). Differences and factors associated with vD deficiency were evaluated using appropriate statistical analysis. vD deficiency in order of magnitude was identified in 62%, 52% and 17% of Mexican, Shanghai and Cincinnati mothers, respectively (*p* < 0.001). In regression analysis, vD supplementation (*p* < 0.01), obesity (*p* = 0.03), season (*p* = 0.001) and sites (*p* < 0.001) predicted maternal vD status. vD deficiency in order of  magnitude was found in 62%, 28%, and 6% of Mexican, Cincinnati and Shanghai infants, respectively (*p* < 0.001). Season (*p* = 0.022), adding formula feeding (*p* < 0.001) and a higher sun index (*p* = 0.085) predicted higher infant vD status. vD deficiency appears to be a global problem in mothers and infants, though the prevalence in diverse populations may depend upon sun exposure behaviors and vD supplementation. Greater attention to maternal and infant vD status starting during pregnancy is warranted worldwide.

## 1. Introduction

Vitamin D (vD) is a prohormone that is synthesized in humans following skin exposure to ultraviolet B radiation in the range of 280–320 nm. In comparison to sunlight, diet provides less than 10% of the body’s vD requirement in unsupplemented individuals [1,2]. Vitamin D is necessary to maintain calcium homeostasis and bone health, and there are increasing reports of its role in innate and autoimmune functions [3]. Vitamin D deficiency is detrimental to the health of mothers and children because of increased risk of osteomalacia in adults and rickets and delayed growth in infants and children [4,5,6]. In addition, vD deficiency or low vD intake has been associated with increased risk of autoimmune diseases in adults and children [7,8] and lower respiratory tract infection in children [9,10,11,12].

Recently, vD deficiency has been reported to be a public health problem worldwide despite abundant sunshine in many countries and the demonstration of the efficacy of vD supplements to prevent vD deficiency [7]. A review of recent studies suggests that vD deficiency is a global problem during pregnancy [13]. The prevalence of serum 25(OH)D levels <50 nmol/L, considered vD deficiency [14], ranges between 33% in the USA [15] and 42% in Canada [16] to 75%–77% in the UK [17] and Finland [18] and from 74% to 98% in India [19], New Zealand [20] and the United Arab Emirates [21]. The high prevalence of vD deficiency in pregnancy raises concern about increased risk of low vD status in mothers and infants after birth and especially the segments that breastfeed, because of the low vD content of breast milk. Furthermore, in view of the drive to increase the prevalence and duration of exclusive breastfeeding and the reported increased risk of rickets in breastfeeding infants [5], the vD status of lactating women and their infants should be of global health concern. The reported few studies appear to indicate that vitamin D deficiency is common in breastfeeding infants, and rickets may represent the tip of the iceberg. Some authors have suggested that vD deficiency may be an under-diagnosed public health problem in breastfeeding mothers and their infants in many countries [5,9], but standardized comparisons of global prevalence are lacking. For example, the cutoff values for vD deficiency, age of study, season of study and methods of assessment of risk factors varied among the studies [9]. The objectives of this study were to compare the prevalence and risk factors for vD deficiency in a cohort of breastfeeding mothers and infants in Shanghai, China, Cincinnati, Ohio, and Mexico City, Mexico, using the same study design and serum vD level measurement in a single center. The mothers and infant dyads were enrolled in the longitudinal Global Exploration of Human Milk study, which was designed to explore the effects of different environments and diets on human milk composition, infant nutrition and health.

## 2. Methods

### 2.1. Subjects

One hundred and twenty mother-infant pairs were enrolled at 4 weeks postpartum in Shanghai and Cincinnati and 125 in Mexico City. The protocol of the parent study was approved by the Review Boards of Fudan Children’s Hospital of Fudan University in Shanghai, China, Cincinnati Children’s Hospital Medical Center in Cincinnati, Ohio, and the National Institute of Medical Sciences and Nutrition in Mexico City, Mexico. The parent study was a longitudinal international cohort study of breastfeeding mother-infant pairs designed to assess the relationship between bioactive factors in human milk and infant growth and health status over the first year of life. All of the mothers delivered singleton infants at term (≥37 weeks) and planned to provide breast milk for at least 3 months postpartum. Mothers with health problems that could interfere with breastfeeding or who delivered prematurely (<37 weeks) were excluded from the study. Healthy mothers were recruited from those who delivered at International Peace Maternity and Child Health Hospital in Shanghai, China, The Christ Hospital in Cincinnati, Ohio, and Gea Gonzalez Hospital in Mexico City, Mexico, between March, 2007, and September, 2008.

The primary outcome of this study is the prevalence and risk factors for vD deficiency (measured by serum 25(OH)D concentration) in mothers at 4 weeks postpartum in Mexico City, latitude 19° N, (*n* = 113), Shanghai, latitude 31° N, (*n* = 112), and Cincinnati, latitude 39° N, (*n* = 119) by season. We also studied a subset of 35 Shanghai, 47 Cincinnati and 45 Mexican infants who were assessed during fall and winter seasons at 26 weeks of life at the 3 sites, because they had serum available for 25(OH)D measurement. Cincinnati infants were multi-racial (white (80%), black (10%) and other racial groups (10%)).

### 2.2. Design

A registered nurse visited family homes at 2 weeks postpartum to enroll the mothers after consent had been obtained. For the parent study, postpartum visits took place at 4 weeks, 13 weeks, 26 weeks, 52 weeks and 108 weeks to complete demographic data and follow-up questionnaires. The data collected prospectively for the purpose of this study included socio-demographics, maternal vD supplementation intake reported during the interview and sunlight exposure behaviors in mothers and infants. Maternal report of vD supplementation to the infant was incomplete in all 3 sites, and therefore, comparisons of vD supplementation of infants at the 3 sites was not available. Infant milk source during the follow-up was documented using standardized questionnaires during weekly phone follow-up interviews. It was, therefore, possible to determine the prevalence of formula usage, which could impact infant’s vD intake and, thus, vD status.

Sun exposure behavior was assessed by recording the duration of direct sun exposure (h/week) in the week prior to the interview. Body surface area (BSA) exposure while outdoors was based on a modified questionnaire [22] for assessing sun exposure to sunlight in adults and infants using a mode of outdoor clothing. For example, exposure of head and neck is assigned an area of 5% for the mother and 14% for the infant. The total percentage of BSA exposed to sunlight was calculated as the sum of the percentage BSA exposed while outdoors associated with all of the subject’s responses. A sun exposure index was calculated by multiplying the percentage of BSA exposed by the hours of exposure to sunlight per week. This index has been shown to correlate with vD status in adults and children [23,24,25].

Blood samples were drawn by venipuncture from the mothers and the infants, and the date of blood collection was recorded. The serum samples were frozen at −80 °C and shipped to the Cincinnati vD research lab from Shanghai and Mexico City sites for evaluation of vD status of mothers and infants. Serum concentrations of 25(OH)D were measured by radioimmunoassay (DiaSorin, Stillwater, MN, USA) in nmol/L, as previously described [22]. The intra- and inter-assay coefficients of variation for 25(OH)D concentration measurement were 4% and 11%, respectively. Serum 25(OH)D concentration <50 nmol/L was defined as deficient [14]; values of 30 to <50 nmol/L were categorized as moderate deficiency, and values <30 nmol/L, consistent with increased risk of rickets or osteomalacia, were categorized as severe deficiency [26,27]. Blood samples were available to measure serum intact parathyroid hormone (PTH) using the immunoradiometric assay (DiaSorin) method in Shanghai and Cincinnati mothers. The normal adult range in the laboratory using this assay is 13–54 pg/mL.

### 2.3. Statistical Analysis

The primary variables for this study were maternal serum 25(OH)D concentrations at 4 weeks postpartum at the 3 sites and the infant serum 25(OH)D concentrations at 26 weeks postpartum. Analysis of variance was used to compare mothers by site and within each season for serum 25(OH)D concentrations and sun exposure index. Infant serum 25(OH)D concentrations during fall and winter seasons at 26 weeks of age by site was compared using ANOVA.

We also compared selected maternal socio-demographic factors, prevalence of vD supplementation and sun exposure behaviors by sites using nonparametric statistical tests. Multivariate multiple regression models with the vD status of the mothers at 4 weeks postpartum and the infants at 26 weeks of age during the fall/winter season as the outcome variable were constructed to control for other potential confounding variables.

## 3. Results

### 3.1. Maternal Results

Table 1 shows the comparison of the maternal baseline characteristics by site. Cincinnati mothers were older (*p* < 0.001), included mothers with higher prevalence of obesity (*p* = 0.0001), four-year college education (*p* = 0.0001) and vD supplementation rate (*p* = 0.0001). The mean sun index was negligible in Shanghai (10.0) and Mexican mothers (0.87) compared with Cincinnati mothers (239.0), *p* < 0.001.

**Figure 1:**
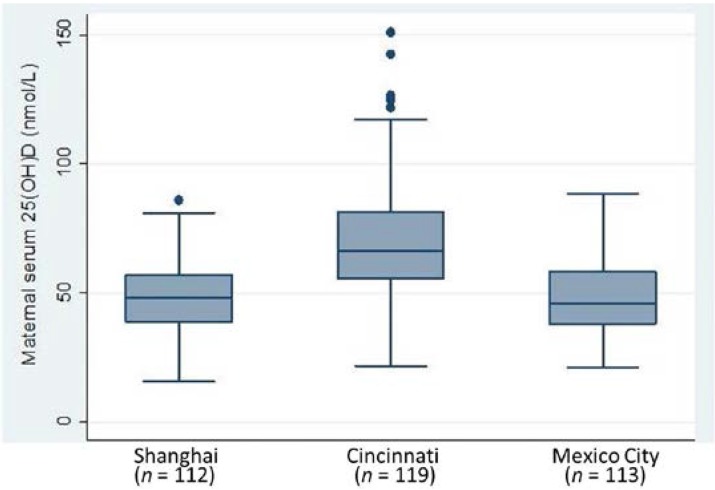
Maternal serum 25(OH)D concentrations, *p* = 0.001 by ANOVA.

At four weeks postpartum, maternal mean serum 25(OH)D concentrations differed (*p* < 0.001) by site (Figure 1). The mean values of 48.6 nmol/L in Shanghai and 48.2 nmol/L in Mexico City were lower than the value of 70.2 nmol/L in Cincinnati. Vitamin D deficiency (serum 25(OH)D <50 nmol/L) in order of magnitude was found in 62%, 52% and 17% of Mexican, Shanghai and Cincinnati mothers, respectively (*p* < 0.001).

**Table 1:** Maternal demography, vitamin D (vD) supplementation and sun index by city.

	Shanghai Latitude 31° N	Cincinnati Latitude 39° N	Mexico City Latitude 19° N	*p*-Value
Age (mean (SD))	29.3 (3.7)	31.5 (5.2)	24.4 (5.6)	0.001
Obese (BMI >30) (*n* (%))	1 (0.8)	33 (28.0)	5 (4.5)	0.0001
Education (*n* (%)), completed 4-y college	69 (57.5)	82 (68.3)	5 (4.2)	0.0001
vD supplementation (*n* (%))	22 (18.3)	94 (87.0)	44 (35.2)	0.0001
Sun index (mean (SD))	10 (24)	239 (301)	0.87 (1.1)	0.001

*p*-values by Fisher’s or the Kruskal–Wallis test.

**Figure 2:**
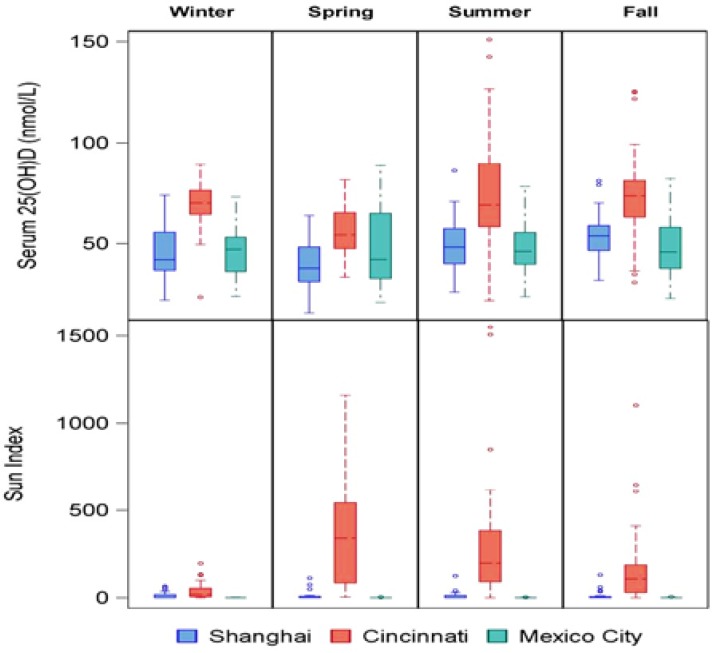
Maternal vD status and sun index by season and site, *p* < 0.001 by ANOVA, comparing mothers by site within each season for vitamin D and sun exposure index.

Figure 2 displays the maternal serum 25(OH)D concentrations and sun indices by sites and season. The sun index varies by season among Cincinnati mothers, but there was lack of seasonal variation among Shanghai and Mexican mothers. Maternal vD status as measured by serum 25(OH)D concentrations showed seasonal variation, with the highest values among Cincinnati mothers within each season.

### 3.2. Infant Results

**Figure 3:**
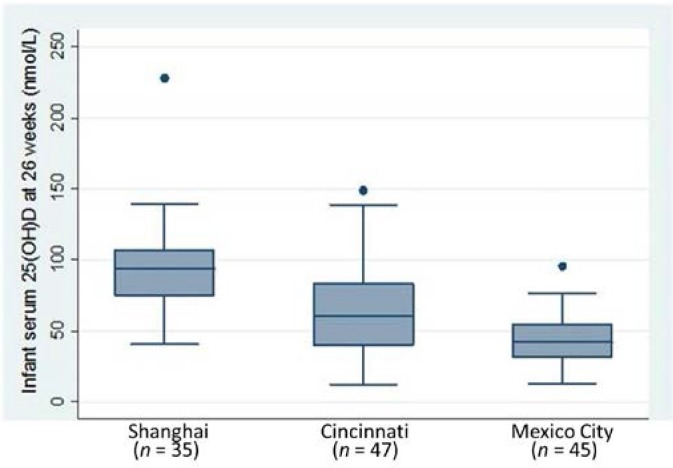
Infant serum 25(OH)D concentrations in fall/winter seasons at 26 weeks of age, *p* < 0.001 by ANOVA.

Comparing infants seen during the fall/winter seasons at 26 weeks of age at the three sites, the mean serum 25(OH)D concentrations were lower in Mexico City (44.0 nmol/L) and Cincinnati (68.3 nmol/L) than Shanghai (95.3 nmol/L), *p* < 0.001 (Figure 3). Vitamin D deficiency in order of magnitude was found in 62%, 28% and 6% of Mexican, Cincinnati and Shanghai infants, respectively (*p* < 0.001). Sunlight exposures during the fall/winter seasons were different by sites and were higher in Shanghai infants at 26 weeks than in Cincinnati and Mexican infants. The duration of sun exposure in hours per week (median, IQR) were 3.0 (1.1, 4.4) *vs.* 2.0 (0.5, 5.3) *vs.* 1.0 (0.3, 1.7) in Shanghai, Cincinnati and Mexican infants, respectively. The median (IQR) percent of BSAs exposed were 26 (14, 26) *vs.* 14 (7, 26) *vs.* 19 (14, 24) in Shanghai, Cincinnati and Mexican infants, respectively. Median (IQR) sun index (BSA × duration of sun exposure) values were 55 (22,104) *vs.* 27 (3.8, 65) *vs.* 21 (7.1, 29.1) in Shanghai, Cincinnati and Mexican infants, respectively. In spring/summer seasons, the infant sun index was higher in the Cincinnati cohort followed by Shanghai and lowest in the Mexico City cohort, but we did not have enough blood for infant serum 25(OH)D measurement in the Shanghai and Mexico City cohorts. Serum 25(OH)D in infants are associated with their sun index, including all infants measured in the fall/winter season (*r* = 0.39, *p* < 0.001) across the three sites. This is also true for infants in the Cincinnati site in the fall/winter season (*r* = 0.37, *p* = 0.01), but not for the other two sites independently.

### 3.3. Categories of vD Deficiency in Mothers and Infants

The different categories of low vD status, moderate (serum 25(OH)D 30 to <50 nmol/L) and severe (serum 25(OH)D <30 nmol/L) deficiency, in mothers and infants differed by site and are shown in Table 2. Overall, less than 4% of the mothers were severely deficient, and the percent of mothers with severe deficiency was over two-fold higher in Mexican mothers than Shanghai and Cincinnati mothers. About half of the Shanghai and Mexican mothers had moderate deficiency compared with 14% of Cincinnati mothers. Overall, 12.5% of the infants evaluated during the fall/winter season at 26 weeks of age were severely deficient. In contrast to maternal findings, the percent of infants with severe and moderate deficiencies were lower in Shanghai compared with Cincinnati and Mexico City cohorts.

**Table 2:** Categories of vitamin D status in mothers and infants.

	All	Shanghai	Cincinnati	Mexico City
Categories				
Mothers * at 4 weeks postpartum	344	112	119	113
n (%) <30 nmol/L	13 (3.8)	3 (2.7)	3 (2.5)	7 (6.2)
n (%) 30 to <50 nmol/L	135 (39.2)	55 (49.1)	17 (14.3)	63 (55.8)
n (%) ≥50 nmol/L	196 (57.0)	54 (48.2)	99 (83.2)	43 (38.0)
Infants * at 26 weeks of age	128	36	47	45
n (%) <30 nmol/L	16 (12.5)	0 (0)	6 (12.8)	10 (22.2)
n (%) 30 to <50 nmol/L	27 (21.0)	2 (5.6)	7 (14.9)	18 (40.0)
n (%) ≥50 nmol/L	85 (66.4)	34 (94.4)	34 (72.3)	17 (37.8)

* *p* ≤ 0.001 by Fisher’s exact test comparing categories of vD status by site.

### 3.4. Factors Independently Associated with vD Status in Mothers and Infants in Regression Analysis

In the mothers, regression analysis with serum 25(OH)D concentration as the primary outcome showed that vD supplement intake (β ± SE, 5.5 ± 2.2, *p* = 0.01) and summer/fall season (β ± SE, 7.3 ± 1.8, *p* < 0.001) were associated with higher maternal serum 25(OH)D concentrations, while obesity (β ± SE, −6.7 ± 3.1, *p* = 0.03) and sites (Shanghai (β ± SE, −19.9 ± 2.8, *p* < 0.001) and Mexico City (β ± SE, −20.8 ± 6, *p* < 0.001)) were associated with low maternal serum 25(OH)D concentrations. Factors evaluated in the model were obesity or prepregnancy, BMI, age, education, season, vD supplementation, sun index and sites. In the Cincinnati cohort only was there a higher sun index, a significant predictor of maternal vD status. In regression analysis, formula feeding (*p* = 0.001), season (*p* = 0.022), sun index (*p* = 0.085) and sites (*p* ≤ 0.001) were independent predictors of higher serum 25(OH)D concentration status in the infants at 26 weeks of age. Factors evaluated in the model were sun index log, percent formula fed, mother’s serum 25(OH)D level, maternal vitamin D deficiency and sites.

### 3.5. Maternal Serum 25(OH)D Concentrations and PTH Relationship in Cincinnati and Shanghai

**Figure 4:**
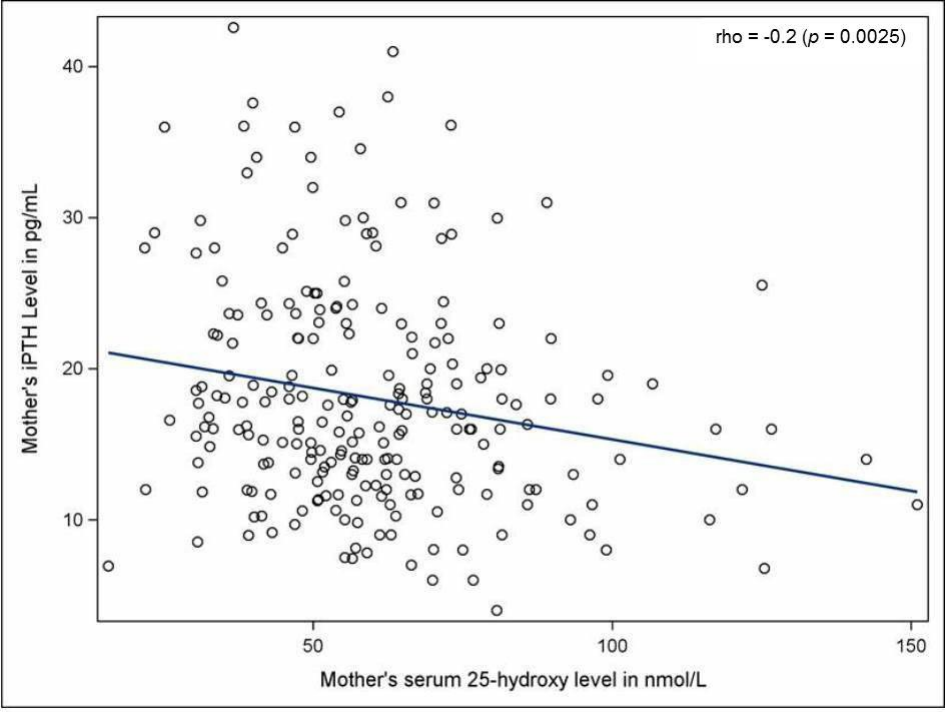
Correlation between parathyroid hormone (PTH) (pg/mL) and 25(OH)D (nmol/L) levels at four weeks post-partum in Shanghai and Cincinnati mothers. Analyzed together, serum 25(OH)D and PTH were negatively correlated.

There was a negative correlation between serum 25(OD) concentrations and PTH concentrations (*r* = −0.2, *p* = 0.002) (Figure 4) in mothers at four weeks postpartum based on the data from Shanghai and Cincinnati. Blood samples were insufficient to measure PTH concentrations in mothers from Mexico City.

## 4. Discussion

In this prospective study of urban population in North America, Latin America and China using the same study design, we found vD deficiency to be common in breastfeeding mothers from Shanghai and Mexico City and less common in mothers in Cincinnati. The mean serum 25(OH)D concentration in lactating mothers in Mexico City (48.2 nmol/L) and in Shanghai (48.6 nmol/L) was lower than the recommended target value of >50 nmol/L [27]. At four weeks postpartum, over 60% of the mothers in Mexico City and half of the mothers in Shanghai were vD deficient ()serum 25(OH)D concentration <50 nmol/L) compared with 17% of mothers in Cincinnati. Most of the vD deficient mothers had moderate vD deficiency (serum 25(OH)D levels of 30 to <50 nmol/L) while 2% of the mothers in Shanghai and Cincinnati and 6.2% of Mexican mothers had severe deficiency (serum 25(OH)D levels <30 nmol/L), which is associated with increased risk of osteomalacia. As expected, there was an inverse relationship between 25(OH)D levels and serum PTH levels, indicating an inadequate vD status in the mothers, which is associated with elevated levels of serum PTH. The degree of severe vD deficiency is significantly lower than previously reported from other countries. Based on previous studies, 61% of breastfeeding mothers from the United Arab Emirates [28], 48% of mothers in India [29] and 46% of breastfeeding mothers from Turkey [30] had severe deficiency (serum 25(OH)D concentration <25 nmol/L), which is associated with increased risk of osteomalacia [27]. Such a low vD status would also predispose breastfeeding infants without vD supplementation to vD deficiency. It thus appears that moderate to severe vD deficiency in early postpartum in breastfeeding mothers is a common problem in many countries and may be under recognized.

In this study, the higher vD status in mothers from Cincinnati was associated with higher sun index and vD supplementation intake compared with mothers in Shanghai and Mexico City. It is of note that the restricted exposure to sunlight during postpartum among Shanghai and Mexican mothers is related to cultural practices in which mothers are restricted from outdoor activities and are cared for by family members. This practice of restricting mothers from outdoor exposure in the immediate postpartum convalescent period (known as “doing the month”) in China has been associated with increased risk of low vD status and rickets in some rural Chinese communities [31]. Recognition of the possible impact of these cultural practices on vD nutrition in postpartum mothers should heighten attention to the need for vD supplementation in such settings. In all of the cohorts from the three sites, vD intake, obesity and season were independent predictors of maternal vD status in multivariate regression analysis. In individual site analysis, high maternal sun index (>500) was associated with high maternal vD status only in the Cincinnati cohort, which attained such a high index. Therefore, differences in the prevalence of these potential risk factors among populations will impact maternal global prevalence, as well as the degree of maternal vD deficiency. For example, in the study from the United Arab Emirates, where severe vD deficiency is more common than in this study, it was found that mothers were more severely sunshine deprived during lactation and had a lower rate of vD supplement intake [28,32].

The vitamin D status of breastfeeding infants who were seen at 26 weeks of age during the fall and winter seasons were lower in the Mexican infants (mean serum 25(OH)D 44 nmol/L) and Cincinnati infants (68.3 nmol/L) than in Shanghai infants (95.3 nmol/L). Vitamin D deficiency, defined as serum 25(OH)D <50 nmol/L, was ten-fold higher in Mexican infants and almost five-fold higher in Cincinnati infants than in Shanghai (62% *vs.* 28% *vs.* 6%, respectively). In addition, severe deficiency (serum 25(OH)D <30 nmol/L) was over two-fold higher in Mexican than in Shanghai and Cincinnati infants. This degree of severe deficiency could theoretically predispose Mexican infants to increased risk of rickets [26,27].

Other recent studies indicate that the prevalence of serum 25(OH)D <30 nmol/L is high and variable worldwide in breastfeeding infants, and lack of sun exposure and vD supplementation have been suggested as contributing factors [9]. Twenty-seven percent of breastfeeding infants in Ioannina in Greece [33], 43%–48% of breastfed infants from New Delhi, India [29,34], and 82% of exclusively breastfed infants in Al Ain, the United Arab Emirates [28], had serum 25(OH)D <25 nmol/L at 3–6 months of age. It therefore appears that moderate to severe vD deficiency is common in sunshine-deprived and unsupplemented breastfed infants, and reports of clinical rickets may not represent the true picture of low vitamin D status in breastfed infants.

Although the mothers and infants in Shanghai were sunshine deprived in the immediate postpartum period, it is of interest that the infants had a higher sun index than the Cincinnati and Mexican infants during the fall/winter season at 26 weeks of age. Clinical experience also suggested that most (80%) of breastfeeding infants in Shanghai would be on vD supplements, because it is encouraged by care providers [35]. Higher sun index, intake from formula feeding and possible high vD supplementation probably contributed to the higher vD status in Shanghai infants. Using available data from the cohorts from the three sites in the regression analysis, a higher sun index and formula feeding, which increased infant vD intake, were predictors of higher infant vD status at 26 weeks of age. In a previous report, which focused on the Cincinnati site, a shorter duration of exclusive breastfeeding was also predictive of vD sufficiency [22].

The strength of the study is that we had data to examine the effect of sun exposure and vD supplement intake on maternal vD status at three international sites using the measurement of 25(OH)D levels in a single center. Our study also had a number of limitations. The measurement of 25(OH)D was at only one time point, and we did not have data to compare mother-infant pairs over time. There was lack of information on the vD status of the infants between birth and 26 weeks of age, which could have provided better longitudinal data on the relationship between feeding pattern and serum 25(OH)D concentration in the infant. We did not have blood samples in the infants for comparison of 25(OH)D concentrations during all four seasons across the three sites. We did not evaluate the role of skin pigmentation, which could contribute to vitamin synthesis and serum 25(OH)D levels, especially in Cincinnati cohorts. Additionally, an important limitation was lack of data on the rate of vD supplement intake in the infants at two of the three sites.

The reported vD supplementation rate in infants in the Cincinnati cohort was only 19%. Other studies from the U.S. have also reported a low vD supplementation rate of 5%–19% in breastfeeding infants [36,37], while two recent studies from Canada found high vD supplementation rates of 80%–98% [38] and 88%–98% in breastfed infants [39]. Advice on vD supplement use from healthcare providers was a positive predictor of supplementation in the Canadian [38,39] and one U.S. [37] study. In general, breastfed infants rely on transplacental transfer of vD, skin synthesis of vD or vD supplementation. However, due to concern about skin cancer, professional organizations recommend that infants avoid sun exposure [40]. Therefore, if breast milk is a major source of feeding in a setting of low maternal vD status and limited sun exposure, awareness among healthcare providers and caregivers of the need for vD supplement intake should be heightened to prevent vD deficiency in breastfeeding infants.

## 5. Conclusions

Vitamin D deficiency is detrimental to the health of mother and infant. From this comparative study using the same study design, it is possible that vD deficiency may be a global health problem in the breastfeeding mother-infant dyad and is related to sun exposure behaviors and vD supplement use between populations. Greater attention to maternal and infant vD status, preferably starting during pregnancy, is warranted worldwide.
